# An exome array study of the plasma metabolome

**DOI:** 10.1038/ncomms12360

**Published:** 2016-07-25

**Authors:** Eugene P. Rhee, Qiong Yang, Bing Yu, Xuan Liu, Susan Cheng, Amy Deik, Kerry A. Pierce, Kevin Bullock, Jennifer E. Ho, Daniel Levy, Jose C. Florez, Sek Kathiresan, Martin G. Larson, Ramachandran S. Vasan, Clary B. Clish, Thomas J. Wang, Eric Boerwinkle, Christopher J. O'Donnell, Robert E. Gerszten

**Affiliations:** 1 Nephrology Division, Massachusetts General Hospital, 55 Fruit Street, Boston, Massachusetts 02114, USA; 2 Endocrinology Division, Massachusetts General Hospital, 55 Fruit Street, Boston, Massachusetts 02114, USA; 3 Metabolite Profiling, Broad Institute of MIT and Harvard, 415 Main Street, Cambridge, Massachusetts 02142, USA; 4 Department of Biostatistics, Boston University School of Public Health, 801 Massachusetts Avenue, Boston, Massachusetts 02118, USA; 5 Human Genetics Center, University of Texas Health Science Center at Houston, 1200 Pressler Street, Houston, Texas 77030, USA; 6 National Heart, Lung and Blood Institute's Framingham Heart Study, 73 Mount Wayte Avenue, Framingham, Massachusetts 01702, USA; 7 Division of Cardiovascular Medicine, Brigham and Women's Hospital, 75 Francis Street, Boston, Massachusetts 02115, USA; 8 Cardiology Division, Massachusetts General Hospital, 55 Fruit Street, Boston, Massachusetts 02114, USA; 9 Population Sciences Branch, National Heart, Lung, and Blood Institute, Bethesda, Maryland 20892, USA; 10 Diabetes Research Center, Massachusetts General Hospital, 55 Fruit Street, Boston, Massachusetts 02114, USA; 11 Center for Human Genetic Research, Massachusetts General Hospital, 185 Cambridge Street, Boston, Massachusetts 02114, USA; 12 Program in Medical and Population Genetics, Broad Institute of MIT and Harvard, 415 Main Street, Cambridge, Massachusetts 02142, USA; 13 Cardiovascular Research Center, Massachusetts General Hospital, 185 Cambridge Street, Boston, Massachusetts 02114, USA; 14 Department of Mathematics and Statistics, Boston University, 111 Cummington Mall, Boston, Massachusetts 02215, USA; 15 Preventive Medicine and Epidemiology, Boston University School of Medicine, 801 Massachusetts Avenue, Boston, Massachusetts 02118, USA; 16 Division of Cardiovascular Medicine, Vanderbilt University Medical Center, 1211 Medical Center Drive, Nashville, Tennessee 37232, USA; 17 Vanderbilt Heart and Vascular Institute, 1211 Medical Center Drive, Nashville, Tennessee 37232, USA; 18 Human Genome Sequencing Center, Baylor College of Medicine, 1 Baylor Plaza, Houston, Texas 77030, USA; 19 Cardiology Section, Boston Veteran's Administration Healthcare, 1400 VFW Parkway, Boston, Massachusetts 02132, USA; 20 Division of Cardiovascular Medicine, Beth Israel Deaconess Medical Center, 185 Pilgrim Road, Boston, Massachusetts 02215, USA

## Abstract

The study of rare variants may enhance our understanding of the genetic determinants of the metabolome. Here, we analyze the association between 217 plasma metabolites and exome variants on the Illumina HumanExome Beadchip in 2,076 participants in the Framingham Heart Study, with replication in 1,528 participants of the Atherosclerosis Risk in Communities Study. We identify an association between *GMPS* and xanthosine using single variant analysis and associations between *HAL* and histidine, *PAH* and phenylalanine, and *UPB1* and ureidopropionate using gene-based tests (*P*<5 × 10^−8^ in meta-analysis), highlighting novel coding variants that may underlie inborn errors of metabolism. Further, we show how an examination of variants across the spectrum of allele frequency highlights independent association signals at select loci and generates a more integrated view of metabolite heritability. These studies build on prior metabolomics genome wide association studies to provide a more complete picture of the genetic architecture of the plasma metabolome.

## Abstract (web summary)

Several GWAS have identified many common variants associated with blood metabolites. Here, the authors use an exome array to identify low frequency, potentially functional variants that impact human metabolism.

## Graphical abstract

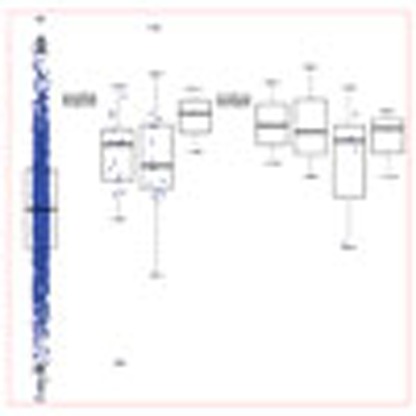

Several independent genome wide association studies (GWAS) have identified dozens of common variants associated with plasma or serum metabolite levels^1^,^2^,^3^,^4^,^5^,^6^,^7^. For example, we recently tested the association between 217 plasma metabolites measured in 2,076 participants of the Framingham Heart Study (FHS) and common variants either directly genotyped using the Affymetrix 500K mapping and 50K gene-focused MIP arrays or imputed from HapMap^5^. This study identified 31 common variants associated with 64 plasma metabolites, and leveraging the family-based structure and rich cardiometabolic phenotyping in FHS, outlined the relative contribution of heritable, environmental and clinical factors to plasma metabolite levels.

The study of low frequency and potentially functional variants, not captured on standard GWAS arrays, has the potential to refine and expand our understanding of the genetic determinants of circulating metabolite levels. Thus, we analyzed the relationship of plasma metabolites in FHS with coding variants captured on the Illumina HumanExome Beadchip, which includes functional exonic variants identified from exome and whole-genome sequencing of over 12,000 individuals^8^. To replicate significant associations, we examined serum metabolite data measured in 1,528 European-American participants in the Atherosclerosis Risk in Communities (ARIC) Study who had been genotyped using the same exome array. Here, we report four genome-wide significant associations, including one identified using single variant analysis and three identified using gene-based testing. In addition, we isolate independent signals at genes highlighted in our prior common variant study and shed light on how variants contribute to metabolite heritability as a function of allele frequency.

## Results

### Single variant analysis highlights *GMPS* and xanthosine

As detailed in the Methods section, we restricted our analysis to the subset of variants that were (1) polymorphic, (2) nonsynonymous, stop-altering or located in a splice site and (3) had a minor allele frequency (MAF) ≤5% (Supplementary Table 1). Eight single variant-metabolite associations reached a significance threshold adjusted for the 81,021 examined variants (*P*<6.2 × 10^−7^ in linear mixed effects models) in FHS. Six of these eight metabolites were also measured in ARIC, and in the replication analysis, the association between a missense variant in *GMPS* (rs61750370) and xanthosine levels remained significant (*P*=2.8 × 10^−7^ in FHS, *P*=6 × 10^−4^ in ARIC, *P*=8.9 × 10^−10^ in meta-analysis) (Table 1). This variant encodes p.Tyr528Ser in guanine monophosphate synthase, the enzyme that catalyzes the oxidation of XMP (the precursor of xanthosine) to guanosine monophosphate (GMP). This finding complements the association between *GMPR*, which encodes the enzyme responsible for the deamination of GMP, and xanthosine identified in our prior GWAS^5^. Because cystathionine was not measured in ARIC, we were unable to replicate the association between a missense variant in *CTH* (rs28941785) and plasma cystathionine levels identified in FHS (Table 1). However, this association reached genome-wide significance in the discovery cohort (*P*=5.4 × 10^−14^) and has clear biologic and clinical underpinnings: the variant encodes p.Thr67Ile in cystathionine gamma-lyase, a cytoplasmic enzyme that converts cystathionine into cysteine, and even more convincing, prior reports have identified homozygosity for this same missense mutation as a cause of cystathioninuria (MIM#219500)^9^,^10^, a benign autosomal recessive disorder characterized by accumulation of plasma cystathionine and increased urinary cystathionine excretion.

### Gene-based analysis identifies three additional associations

Because the number of study participants harbouring any single low-frequency or rare variant is limited, we performed gene-based burden tests to identify additional locus-metabolite associations. These gene-based tests can improve the power to detect associations compared to single variant tests, provided that multiple variants within the gene are causal^11^. To that end, we restricted our analysis to polymorphic variants that are predicted to be damaging (see Methods section). We tested up to 13,008 genes with at least two damaging variants and applied a significance threshold adjusted for the number of genes examined (*P*<3.8 × 10^−6^ in linear mixed effects models). Using this approach, we identified six additional gene-metabolite associations in FHS. Four of these six metabolites were also measured in ARIC, and in the replication analysis, three of the associations remained significant (Table 2), with all three at established human disease loci: *HAL* and histidine (*P*=2.2 × 10^−15^ in FHS, *P*=6 × 10^−5^ in ARIC, *P*=1.4 × 10^−10^ in meta-analysis)—mutations in *HAL* are known to cause the autosomal recessive metabolic disorder histidinemia (MIM # 609457)^12^; *PAH* and phenylalanine (*P*=2 × 10^−11^ in FHS, *P*=5.7 × 10^−6^ in ARIC, *P*=5.9 × 10^−11^ in meta-analysis)—mutations in *PAH* cause the autosomal recessive inborn error of metabolism phenylketonuria (MIM#612349); and *UPB1* and ureidopropionate (*P*=9.8 × 10^−7^ in FHS, *P*=3 × 10^−3^ in ARIC, *P*=3.4 × 10^−8^ in meta-analysis)—mutations in *UPB1* are the cause of the autosomal recessive disorder Beta-ureidopropionase deficiency (MIM # 60667).

**Figure 1:**
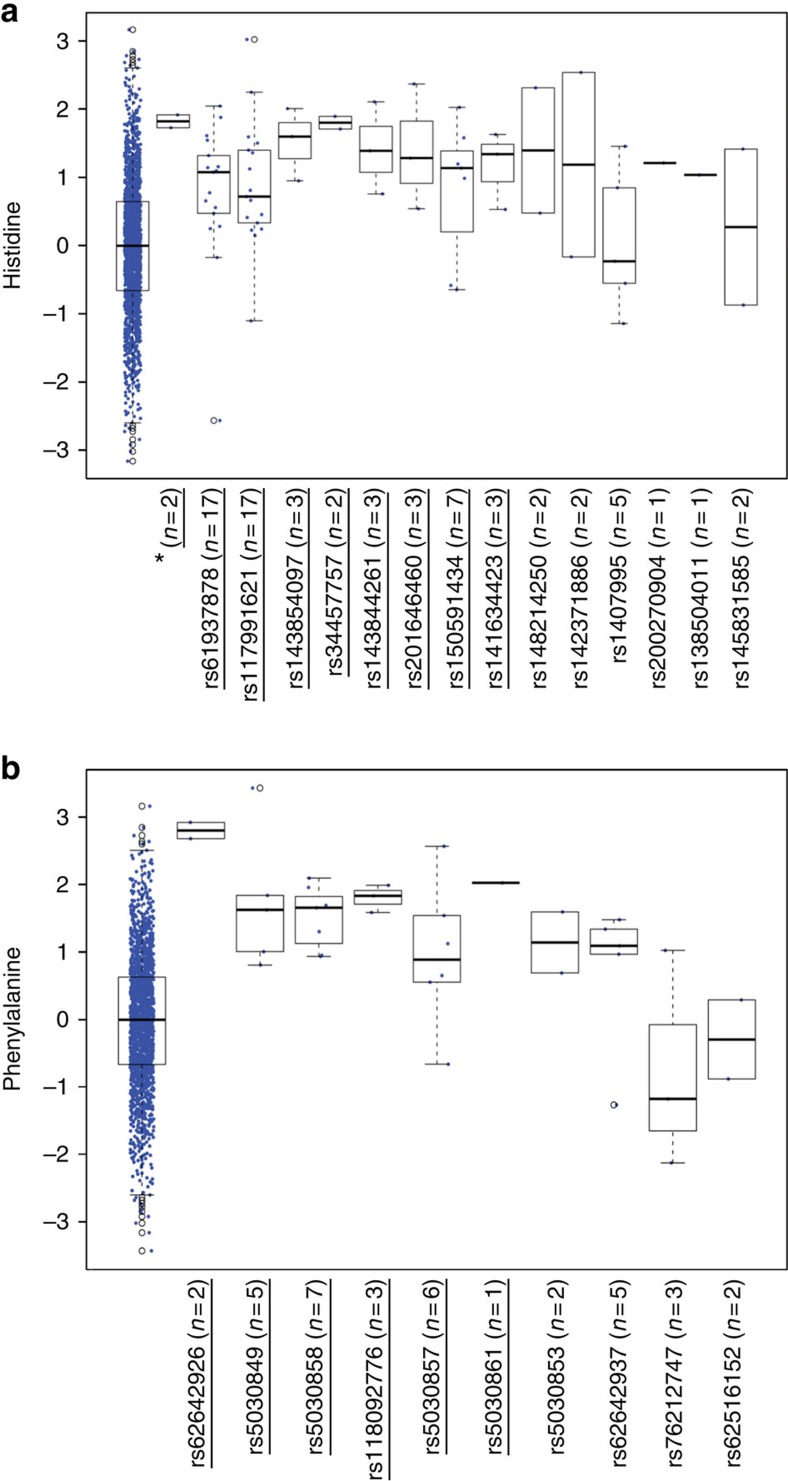
Box plot visualization of gene-based associations at *HAL* and *PAH* in FHS. (**a**) Plasma histidine levels and damaging mutations in *HAL* and (**b**) Plasma phenylalanine levels and damaging mutations in *PAH*, with corresponding rs numbers on *x* axis. Each data point represents an individual's plasma metabolite level (standardized along the *y* axis). Farthest left box plots show metabolite levels among individuals with no damaging mutations. The lines in the boxes represent median metabolite levels; the lower and upper boundaries of the boxes represent the 25th and 75th percentiles, respectively; the lower and upper whiskers represent the minimum and maximum values, respectively. *compound heterozygotes at rs61937878 and rs140799551. SNPs with *P*<0.05 are underlined; *P*-values derived from linear mixed effects models.

A more detailed variant-level examination of the gene-based findings in FHS is instructive. As shown in Table 2 and Fig. 1, individual damaging mutations with at least a nominal metabolite association all had the same direction, and similar magnitude, of effect. Whereas the most common of these variants in *HAL* (Fig. 1a) was found in 19 individuals in FHS, a total of 57 individuals had mutations at any of these 8 variants (2 individuals were compound heterozygotes with mutations at both rs61937878 and rs140799551). Similarly, 6 distinct coding variants in *PAH* predicted to be damaging had at least a nominal association with plasma phenylalanine levels (Fig. 1b) in FHS; whereas the most common of these variants was found in 7 individuals, a total of 24 individuals had any one of these variants (none had more than 1). For *UPB1*, the association with ureidopropionate was driven primarily by a single splice variant.

### Independent common and rare variant associations

We merged the exome chip and common variant data sets in FHS and performed conditional analyses in order to test whether the exome array is able to identify independent signals at loci highlighted by our prior metabolomics GWAS. We restricted our analysis to exome variants not captured in our prior GWAS and identified three coding variants that replicated prior locus-metabolite associations at a genome-wide significant threshold in linear mixed effects models. Results of conditional analyses, whereby exome array variants were adjusted for previously identified noncoding common variants and vice versa, are shown in Table 3. At *DMGDH*, rs145258663 encodes p.Leu300Phe and is independent of the intronic GWAS variant rs248386 (*P*_conditional_ for association with dimethylglycine=1.9 × 10^−9^, *r*^2^=0.031). At *PRODH*, rs5747933 encodes p.Thr167Asn and is independent of the intronic GWAS variant rs2078743 (*P*_conditional_ for association with proline=3.4 × 10^−10^, *r*^2^=0.001). By contrast, the association between rs3135506, which encodes p.Ser19Trp in *APOA5*, and diacylglycerol (DAG) 36:2 was no longer significant after adjusting for the intronic GWAS variant rs964184 (*P*_conditional_=4.9 × 10^−2^, *r*^2^=0.39). Similarly, the common variant signal at this locus was abrogated after adjusting for the coding variant (*P*_conditional_=7.7 × 10^−5^). Given the plausible functional effect at rs3135506, predicted to be damaging by dbNSFP^13^, these data raise the possibility that it is the causal variant that underlies the association between the APOA1/C3/A4/A5 locus and DAG 36:2.

### Interindividual metabolite variation and allele frequency

**Figure 2:**
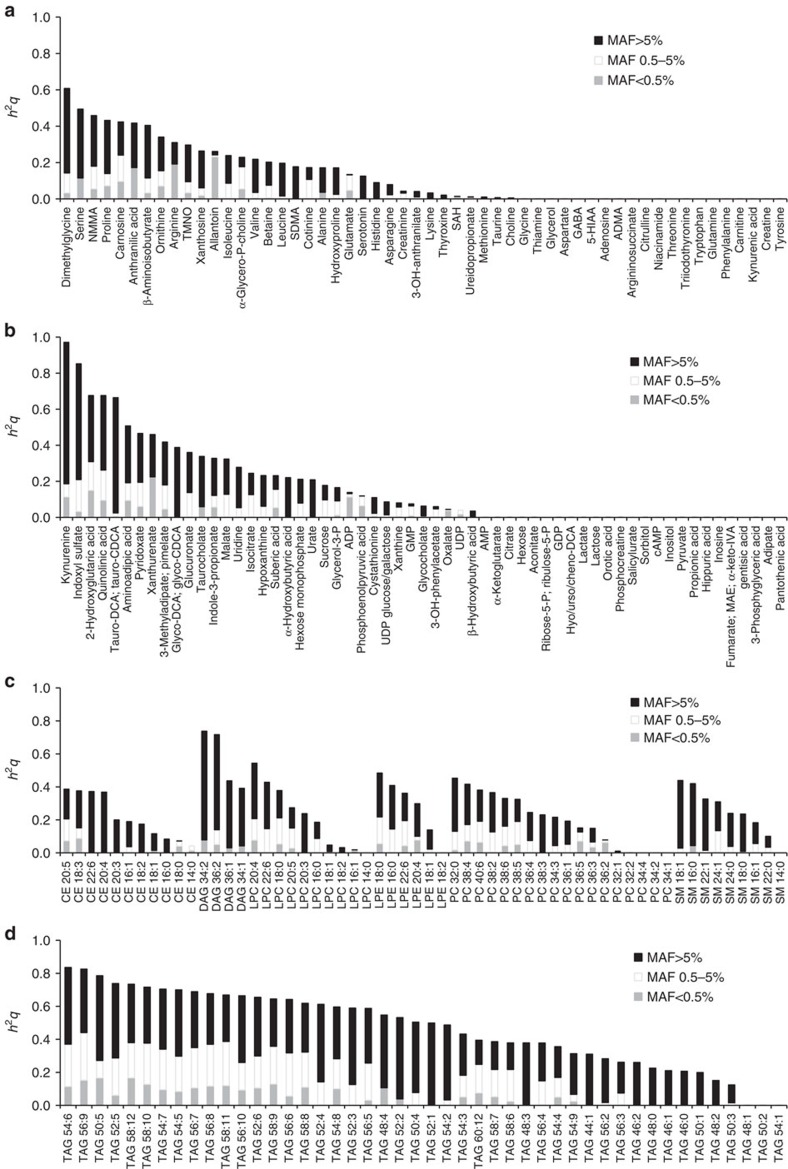
Interindividual variability and allele frequency in FHS. Estimates of metabolite variance explained by SNPs captured across the common variant and exome variant arrays are shown for (**a**) positively charged polar analytes, (**b**) negatively charged polar analytes, (**c**) positively charged lipids, not including TAGs, and (**d**) TAGs. For each metabolite, the relative contribution for SNPs of MAF <0.5%, 0.5–5% and >5% are shown. See Supplementary Table 2 for complete results and explanation of abbreviations.

Because we have now integrated metabolite data with both common variant and exome variant arrays in the same 2,076 individuals, we next estimated the proportion of interindividual metabolite variation captured across a broad range of allele frequency (Fig. 2; Supplementary Table 2). Looking across all non-redundant single nucleotide polymorphisms (SNPs) captured across the two arrays, we confirmed that for many metabolites, a substantial fraction of metabolite variability is heritable^4^,^5^,^14^. When SNPs were then binned on the basis of allele frequency, we found that for many metabolites, low frequency (MAF 0.5–5%) and rare (MAF<0.5%) variants made moderate contributions to overall heritability, although in general less than the contribution made by common variants (MAF>5%).

## Discussion

To date, common variant association studies have shown that many loci have relatively large effect sizes on circulating metabolite levels, as compared to GWAS for common diseases and most risk factor phenotypes. As a result significant findings have emerged from samples as small as 284 subjects^15^. Many of these loci encode enzymes or transporters directly involved with the given metabolite's disposition, providing a strong biologic basis for both the strength and veracity of their associations. In the present study, we extend our analysis of the genetic determinants of plasma metabolite levels in the FHS from an examination of common variants to include coding variants captured on an exome array, followed by replication in ARIC. By definition, statistical significance at this lower end of allele frequency requires loci to have very large effect sizes. Indeed, a major theme of our findings is the recapitulation, and in some cases extension, of variants implicated in Mendelian disorders of human metabolism.

Our data demonstrate significant associations between variants in *CTH*, *HAL*, *PAH* and *UPB1*, and plasma cystathionine, histidine, phenylalanine, and ureidopropionate levels, respectively. Mutations in each of these genes are established causes of autosomal recessive disorders characterized by the defective catabolism and subsequent plasma accumulation of these metabolites. With a MAF of 1% the association between a mutation in *CTH* and plasma cystathionine levels could be detected in single variant analysis. By contrast, the association between the less common mutations in the other genes and their respective metabolites were detected with gene-based testing. Given widespread newborn screening for phenylketonuria, dozens of mutations in the *PAH* locus have now been identified, and indeed the three variants most strongly associated with phenylalanine in our data have been extensively reported in the literature. By contrast, the missense mutation at rs118092776, which encodes p.Arg53His has not been published (it has been catalogued in an online database)^16^. For *UPB1*, the mutation associated with ureidopropionate levels has only been described in one patient in the literature^17^. Similarly, whereas mutations in *HAL* are known causes of histidinemia, the mutations we highlight in association with plasma histidine levels in FHS are novel. Prior reports have identified seven other mutations in *HAL* as either a cause of histidinemia or associated with plasma histidine levels, including four in Japanese individuals^18^ and three among African Americans^19^. Thus taken together, our results illustrate how population-based metabolomic studies are able to supplement decades of biochemical and genetic studies to confirm and potentially even expand the list of coding variants that underlie human disease.

Compared to studies of complex diseases, the study of quantitative phenotypes—in some cases immediately downstream of gene function—can dramatically lower the sample size required to detect statistically significant associations. Nevertheless, the sample sizes examined in FHS and ARIC constrain our power to detect weak associations, a limitation that is mitigated but not eliminated by gene-based testing. It is likely that numerous biologically important associations did not reach statistical significance because of limited effect size and/or limited allele frequency. For example, using established disease loci as a positive control, we highlight the examples of types I and II citrullinemia (MIM#215700, #603859), which are caused by mutations in *ASS1* and *SLC25A13*, respectively. The most commonly reported mutation in type I citrullinemia, rs121908641, demonstrated a trend for association with higher citrulline levels (*β*=1.20, *P*=0.09), despite being present as a single copy in only two individuals in FHS. In *SLC25A13*, we identified a loss-of-function splice mutation at rs150021522 nominally associated with citrulline (*β*=1.33, *P*=0.02). This mutation, present in three individuals in FHS, has not been reported in the literature, perhaps because type II citrullinemia is primarily described in East Asian populations^20^. In order to maximize the utility of our data set, we have made complete exome array results for each of the 217 metabolites surveyed by our platform, as well as complete results of our gene-based analyses, publicly available. We believe that this will facilitate interrogation of the genetic determinants of select metabolites of interest, including biologically meaningful associations of modest statistical significance herein (but potentially of high statistical significance in hypothesis-driven analyses). Further, we note that this data can be queried in conjunction with the catalogue of associations between commons variants and metabolites that we have already published^5^, thus providing a resource for metabolomics research across a broad range of allele frequency.

In our analysis of the estimated proportion of interindividual metabolite variation captured by SNPs across both common variant and exome arrays, we found that low frequency and rare variants made moderate contributions to overall heritability. Although a larger sample size would be required to capture the full contribution of rare variants to metabolite heritability^21^, these findings are consistent with a recent whole-genome sequence-based analysis demonstrating that common variation contributes more to high-density lipoprotein cholesterol heritability than rare variation^22^, We note, however, that our analyses need to be interpreted with caution, as many point estimates for total interindividual variation explained by genetic factors had relatively wide confidence intervals (Supplementary Table 2). Nevertheless, our examination of two genotyping arrays applied to a uniform population demonstrates the heterogeneous relationship between allele frequency and explained variance across 217 different phenotypes.

In summary, we have extended our prior study of the common genetic determinants of plasma metabolites to now include an analysis of lower frequency coding variants sequenced using an exome array. Notably, metabolites were measured using different LC–MS platforms in discovery and replication, to our knowledge the first time this has been done in a study of the genetic determinants of the metabolome, providing increased confidence in our significant findings. By integrating GWAS and exome array data, we also highlight independent association signals at select loci and provide a more granular view of metabolite heritability. Finally, all of our single variant and gene-based analyses have been made publicly available as a resource to the scientific community. Future efforts will seek to examine larger data sets, both in regards to the number of individuals genotyped and the number of metabolites assayed.

## Methods

### Study sample

The FHS Offspring cohort is a prospective, observational, community-based cohort^23^. These children of the initial FHS participants and their spouses were recruited in 1971 and have been followed with serial examinations. A total of 2,076 Offspring participants who attended the fifth examination (1991–1995) and underwent metabolite profiling and exome array genotyping were included in this analysis (Supplementary Table 3). All participants provided informed consent and the study protocol was approved by the Boston University Medical Center IRB.

The ARIC study is a prospective cohort originally designed to assess risk factors for cardiovascular disease in the general population^24^. A total of 15,792 men and women age 45–64 years old were recruited from four communities (Forsyth County, North Carolina; Jackson, Mississippi; northwest suburbs of Minneapolis, Minnesota; and Washington County, Maryland) in 1987–89. Participants were mostly white in the Minneapolis and Washington County sites, white and African-American in Forsyth County, while only African-American individuals were recruited in Jackson. After the baseline examination, participants were invited for four follow-up visits in 1990–92, 1993–95, 1996–98 and 2011–13. Metabolite profiling was performed on serum samples obtained at baseline, between 1987 and 1989. The ARIC study has been approved by the Institutional Review Board at the University of Minnesota, Johns Hopkins University, Wake Forest University, University of North Carolina, University of Texas Health Sciences Center at Houston, and University of Mississippi Medical Center and participants provided written informed consent.

### Metabolite profiling

Blood samples were collected after an overnight fast, immediately centrifuged and stored at −80 °C until assayed.

For FHS samples, metabolites were measured at the Broad Institute. For amino acids, amino acid derivatives, urea cycle intermediates, nucleotides and other positively charged polar metabolites, 10 μl of plasma were extracted in nine volumes of 74.9:24.9:0.2 v/v/v acetonitrile/methanol/formic acid^25^. After centrifugation, supernatants underwent chromatography on a 150 × 2.1 mm Atlantis HILIC column (Waters); mobile phase A: 10 mM ammonium formate and 0.1% formic acid, v/v; mobile phase B: acetonitrile with 0.1% formic acid, v/v. The column was eluted isocratically with 5% mobile phase A for one minute followed by a linear gradient to 60% mobile phase A over 10 min. MS analyses were carried out using electrospray ionization (ESI) and multiple reaction monitoring (MRM) scans in the positive ion mode. The ion spray voltage was 4.5 kV and the source temperature was 425 °C. For the measurement of organic acids, sugars, bile acids and other negatively charged polar metabolites, 30 μl of plasma were extracted with the addition of four volumes of 80:20 v/v methanol/water^5^. After centrifugation, supernatants underwent chromatography on a 150 × 2 mm Luna NH_2_ column (Phenomenex); mobile phase A: 20 mM ammonium acetate, 20 mM ammonium hydroxide; mobile phase B: 10 mM ammonium hydroxide in 25:75 methanol/acetonitrile v/v. The column was eluted isocratically with 10% mobile phase A for 1 min followed by a linear gradient to 100% mobile phase A over 9 min. MS data were acquired using a 5500 QTRAP triple quadrupole mass spectrometer (AB SCIEX) using ESI and MRM in the negative ion mode. The ion spray voltage was −4.5 kV and the source temperature was 500 °C. For lipids, including lysophosphatidylcholines, lysophosphatidylethanolamines, phosphatidylcholines, sphingomyelins, cholesterol esters, DAGs and triacylglyerols, 10 μl of plasma were extracted in 190 μl of isopropanol^26^. After centrifugation, supernatants were separated by reverse phase chromatography using a 150 × 3 mm Prosphere HP C4 column (Grace); mobile phase A: 95:5:0.1 10 mM ammonium acetate/methanol/acetic acid, v/v/v; mobile phase B: 99.9:0.1 methanol/acetic acid, v/v. The column was eluted isocratically with 80% mobile phase A for 2 min followed by a linear gradient to 20% mobile phase A over 1 min, a linear gradient to 0% mobile phase A over 12 min, then 10 min at 0% mobile phase A. MS analyses were carried out using ESI and Q1 scans in the positive ion mode. Ion spray voltage was 5 kV, and source temperature was 400 °C. For each lipid analyte, the first number denotes the total number of carbons in the lipid acyl chain(s), and the second number (after the colon) denotes the total number of double bonds in the lipid acyl chain(s).

For ARIC samples, metabolites were measured using fasting serum, sampled at the baseline ARIC examination. Following receipt at Metabolon Inc. (Durham, NC, USA), untargeted gas chromatography-mass spectrometry and liquid chromatography-mass spectrometry-based metabolomic quantification protocols were used to detect and quantify metabolites^27^,^28^. Compounds were identified by comparison to an in-house generated authentic standard library that includes retention time, molecular weight, preferred adducts, in-source fragments and associated fragmentation spectra of the intact parent ion.

### Exome array

Genotyping was performed using the Illumina HumanExome BeadChip, which captures putative functional exonic variants selected from over 12,000 individual exome and whole-genome sequences. In order to be included, nonsynonymous variants had to be observed two or more times in at least two datasets, and stop-altering and splice variants had to be observed two or more times in at least two datasets. In addition to nonsynonymous, stop-altering and splice variants, additional array content included tags for previously described GWAS hits, ancestry informative markers, random synonymous SNPs, mitochondrial SNPs and human leukocyte antigen tags. In sum, >240,000 variants were included on the exome array. Of these, 109,911 variants were polymorphic in the FHS sample, and a further subset of 81,021 was nonsynonymous, nonsense, or located in a splice site and had a MAF≤5% (Supplementary Table 1). Details on the exome array design can be found at: http://genome.sph.umich.edu/wiki/Exome_Chip_Design.

### Statistical analysis

Due to right-skewed distributions of metabolite levels and differences in scaling, genetic analyses were conducted using normalized residuals of metabolite levels, adjusted for age and sex. The association of genetic variants and metabolite concentrations was tested using linear mixed effects models to accommodate pedigree structure under an additive genetic model. Population stratification was accounted for by adjusting for PC1 if *P*<0.0001, and the final genomic control parameter lambda was between 0.92 and 1.11 with a median of 1.02 for all analyses.

#### Single variant analysis

Analyses were performed using the R GWAF package^29^. For discovery, results were considered significant at a significance threshold adjusted for the number of loci examined, that is, adjusted for the 81,021 polymorphic variants with MAF≤5% that were nonsynonymous, stop-altering or located in a splice site (*P*<6.2 × 10^−7^). For replication, results were considered significant if (1) they reached a significance threshold adjusted for the number of associations examined (*P*<8 × 10^−3^) and (2) following meta-analysis across the discovery and replication cohorts, the *P* value of association reached genome-wide significance (*P*<5 × 10^−8^).

#### Gene-based analysis

The effects of single variant association within a gene were aggregated by summing up the score statistics using the collapsing method in Li and Leal^11^. A variant was considered damaging if it is (1) a stop gain/loss, (2) splice altering or (3) missense and predicted to be damaging by 2 of the 4 algorithms in dbNSFP (Mutation Taster, Polyphen 2 HDIV, SIFT, LRT)^13^. The analysis was carried out using the R seqMeta package across a total of 13,008 genes, and the significance threshold in discovery was adjusted for the number of genes examined (*P*<3.8 × 10^−6^). For replication, results were considered significant if (1) they reached a significance threshold adjusted for the number of associations examined (*P*<1.2 × 10^−2^) and (2) following meta-analysis across the discovery and replication cohorts, the *P* value of association reached genome-wide significance (*P*<5 × 10^−8^).

#### Meta-analysis

Single variant meta-analysis was carried out using the inverse variance weighted method: the meta-analysis beta was the sum of betas from the two cohorts weighted by the inverse of respective variances. To combine the gene-based results from the two cohorts, meta-analysis of each single variant was done first, then the single variant meta-analysis statistics were combined using the method by Pan *et al.*^30^ that accounts for linkage disequilibrium to form a test statistic for each gene.

#### Conditional analysis

SNPs with significant metabolite associations identified in our prior GWAS were included as covariates in the linear mixed effect model of single variant analyses to examine whether the association is attenuated by adjusting for known associations^5^. Similarly, SNPs identified in select single variant analyses were included as covariates in the linear mixed effect model of prior GWAS findings to examine whether the association is attenuated by adjusting for the new coding variant.

#### Heritability

Data on common variant associations from our prior GWAS^5^, which conducted genotyping using the Affymetric 500 K mapping array, was pooled with data acquired herein using the Illumina HumanExome BeadChip. We then examined the estimated proportion of interindividual metabolite variation attributable to all non-redundant, polymorphic SNPs across these two arrays, and subsets of these SNPs binned on the basis of allele frequency (MAF<0.5%, MAF 0.5–5%, MAF>0.5%), using GCTA software^31^. In brief, the genetic relationship between each pair of individuals was estimated as the correlation of genotypes across all SNPs under evaluation. Variance explained by this genetic relationship matrix was estimated in a linear mixed effects model using a subset of unrelated individuals. The quantitative traits loci heritability of all SNPs under evaluation was estimated as the ratio of variance explained by this genetic relationship matrix to total phenotype variance.

### Data availability

Data on singled variant and gene-based results for all metabolites measured in FHS have been deposited in the database of Genotypes and Phenotypes (dbGaP) under accession code phs000007.v28.p10. All other data is available within the manuscript or from the authors upon request.

## Additional information

**How to cite this article:** Rhee, E. P. *et al.* An exome array study of the plasma metabolome. *Nat. Commun.* 7:12360 doi: 10.1038/ncomms12360 (2016).

## Supplementary Material

Supplementary Information Supplementary Tables 1-3

## Figures and Tables

**Table 1:** Single variants associated with plasma metabolites.

**Trait**	**Variant information**						**Discovery cohort (FHS)** ***N*****=2,076**			**Replication cohort (ARIC)** ***N*****=1,528**			**Meta-analysis**	
	**Gene**	**SNP**	**Variant**	**Chr**	**Position**	**Major/minor allele**	**MAF**	**Beta**	***P*-value**	**MAF**	**Beta**	***P*-value**	**Beta**	***P*-value**
Cystathionine	*CTH*	rs28941785	p.Thr67Ile	1	70881670	C/T	0.01	1.34	5.5 × 10^−14^	Metabolite not measured				
Xanthosine	*GMPS*	rs61750370	p.Tyr528Ser	3	155649576	A/C	0.01	0.95	2.8 × 10^−7^	0.01	0.61	6 × 10^−4^	0.78	8.9 × 10^−10^

ARIC, atherosclerosis risk in communities study; FHS, Framingham Heart Study; SNP, single nucleotide polymorphism.

*P*-values derived from linear mixed effects models.

**Table 2:**
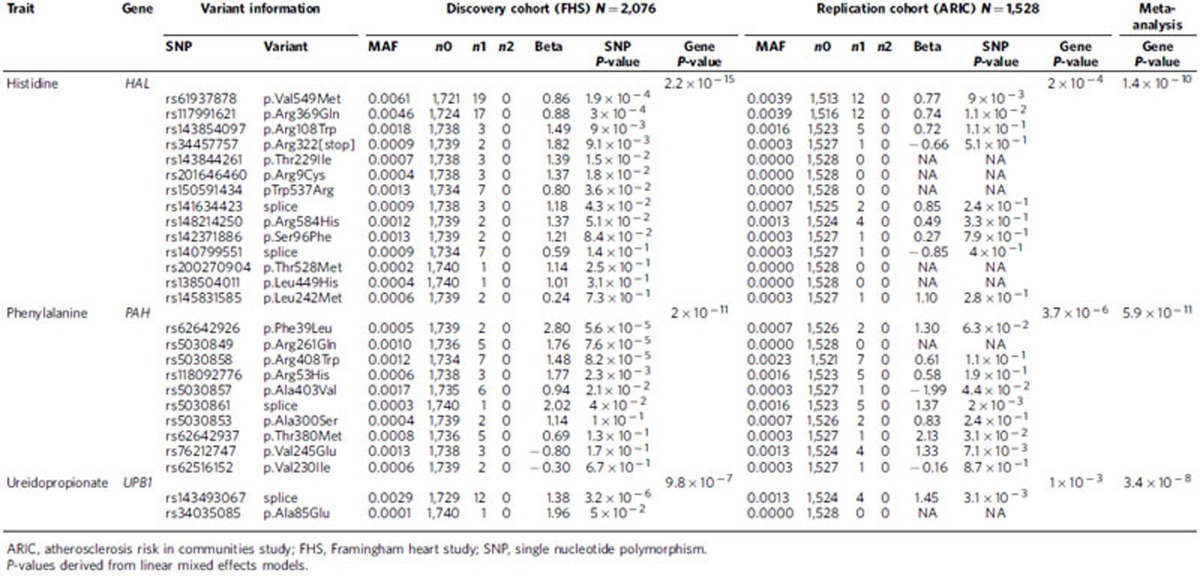
Gene-based associations with plasma metabolites.

**Table 3:** New exome variants at loci previously identified by GWAS.

**Metabolite**	**Gene**	**Chr**	**Position**	**SNP**	**SNP type**	**MAF**	**Major/minor allele**	***P*-value**	**Conditioning SNP**	***P***_**conditional**_
Dimethylglycine	*DMGDH*	5	78340223	rs145258663	Nonsynonymous	0.005	G/A	7 × 10^−22^	rs248386	1.9 × 10^−9^
		5	78365983	rs248386	Intronic	0.18	C/A	6.6 × 10^−33^	rs145258663	4.5 × 10^−19^
Proline	*PRODH*	22	18910355	rs5747933	Nonsynonymous	0.049	G/T	2.3 × 10^−12^	rs2078743	3.4 × 10^−10^
		22	17346859	rs2078743	Intronic	0.090	G/A	2.2 × 10^−14^	rs5747933	3.7 × 10^−12^
DAG 36:2	*APOA5*	11	116662407	rs3135506	Nonsynonymous	0.062	G/C	2.7 × 10^−8^	rs964184	4.9 × 10^−2^
		11	116154127	rs964184	Intronic	0.14	C/G	1.3 × 10^−11^	rs3135506	7.7 × 10^−5^

DAG, diacylglycerol, SNP, single nucleotide polymorphism.

*P*-values derived from linear mixed effects models.
