# Supplementary material for: An exome array study of the plasma metabolome
Source: Nat Commun. 2016 Jul 25;7:12360. doi: 10.1038/ncomms12360 (PMC4962516; doi:10.1038/ncomms12360)
Supplement: Supplementary Information — Supplementary Tables 1-3 [file ncomms12360-s1.pdf]

Supplementary Table 1 Polymorphic exome array variants in 2,076 individuals in FHS

	MAF			
	**<0.5**	**0.5 to 5**	**>5**	**Total**
Polymorphic	69880	14450	25581	109911
Nonsynonymous	67924	13097	11612	92633
Damaging	34267	5321	2413	42001
Loss-of-function	4249	635	599	5483

Supplementary Table 2 Proportion of interindividual variation and allele frequency

Metabolite	Figure 2 Abbreviation	total h^2q	SE	*P*	Percent of total h^2q MAF < 0.5%	MAF 0.5-5%	MAF > 5%
3-hydroxyanthranilic acid	3-OH-anthranilate	0.04	0.18	0.4	0.0	25.9	74.1
5-hydroxyindoleacetic acid	5-HIAA	1.0E-06	0.16	0.5	33.3	33.3	33.3
adenosine		1.0E-06	0.21	0.5	33.3	33.3	33.3
alanine		0.17	0.21	0.3	20.8	0.0	79.2
allantoin		0.27	0.20	0.06	85.0	3.6	11.4
alpha-glycerophosphocholine	α-glycero-P-choline	0.22	0.18	0.03	24.7	53.9	21.3
anthranilic acid		0.42	0.22	0.04	40.9	0.0	59.1
arginine		0.31	0.22	0.1	62.1	0.0	37.9
argininosuccinate		1.0E-06	0.39	0.5	33.3	33.3	33.3
asparagine		0.08	0.16	0.3	0.9	28.3	70.7
aspartate		1.0E-06	0.24	0.5	0.0	0.0	100.0
asymmetric dimethylarginine	ADMA	1.0E-06	0.12	0.5	33.3	33.3	33.3
beta-aminoisobutyric acid	β-aminoisobutyric acid	0.40	0.20	0.002	8.4	20.2	71.4
betaine		0.20	0.16	0.02	3.0	34.5	62.5
carnitine		1.0E-06	0.14	0.5	0.0	0.0	100.0
carnosine		0.41	0.40	0.1	23.4	34.8	41.8
choline		4.9E-03	0.09	0.5	2.1	97.9	0.0
citrulline		1.0E-06	0.18	0.5	0.0	100.0	0.0
cotinine		0.17	0.19	0.2	0.0	62.6	37.4
creatine		1.0E-06	0.19	0.5	0.0	34.4	65.6
creatinine		0.04	0.11	0.3	7.0	64.1	28.9
dimethylglycine		0.60	0.23	0.0003	5.5	18.1	76.3
gamma-aminoisobutyric acid	GABA	1.0E-06	0.20	0.5	100.0	0.0	0.0
glutamate		0.13	0.19	0.3	36.0	63.4	0.6
glutamine		1.0E-06	0.14	0.5	0.0	100.0	0.0
glycerol		1.0E-06	0.16	0.5	0.0	0.0	100.0
glycine		1.0E-06	0.15	0.5	0.0	0.0	100.0
histidine		0.09	0.16	0.3	6.7	0.0	93.3
hydroxyproline		0.17	0.18	0.2	0.0	14.6	85.4
isoleucine		0.24	0.20	0.1	0.0	35.6	64.4
kynurenic acid		1.0E-06	0.18	0.5	0.0	35.4	64.6
leucine		0.20	0.19	0.2	0.0	8.2	91.8
lysine		0.03	0.12	0.4	2.2	0.0	97.8
methionine		0.01	0.11	0.5	0.2	0.0	99.8
NG-monomethyl-arginine	NMMA	0.46	0.22	0.005	12.1	26.9	61.0
niacinamide		1.0E-06	0.11	0.5	33.3	33.3	33.3
ornithine		0.34	0.20	0.03	20.9	24.6	54.5
phenylalanine		1.0E-06	0.14	0.5	0.0	0.0	100.0
proline		0.43	0.21	0.007	16.8	15.3	67.8
S-adenosylhomocysteine	SAH	0.01	0.13	0.5	100.0	0.0	0.0
serine		0.50	0.24	0.02	23.2	0.0	76.8
serotonin		0.13	0.19	0.3	0.0	0.0	100.0
symmetric dimethylarginine	SDMA	0.18	0.23	0.5	0.0	0.0	100.0
taurine		0.01	0.12	0.5	0.0	12.5	87.5
thiamine		1.0E-06	0.13	0.5	0.0	0.0	100.0
threonine		1.0E-06	0.08	0.5	25.3	74.7	0.0
thyroxine		0.02	0.10	0.4	0.0	22.1	77.9
triiodothyronine		1.0E-06	0.15	0.5	33.3	33.3	33.3
trimethylamine-N-oxide	TMNO	0.30	0.17	0.002	11.5	20.3	68.2
tryptophan		1.0E-06	0.12	0.5	0.0	100.0	0.0
tyrosine		1.0E-06	0.15	0.5	0.0	0.0	100.0
ureidopropionic acid		0.01	0.14	0.5	0.0	82.6	17.4
valine		0.22	0.20	0.09	2.2	14.0	83.8
xanthosine		0.27	0.20	0.05	7.3	15.6	77.1
CE 14:0		0.04	0.13	0.4	37.0	63.0	0.0
CE 16:0		0.08	0.15	0.3	0.0	4.3	95.7

CE 16:1	0.19	0.17	0.07	0.0	19.9	80.1
CE 18:0	0.07	0.15	0.3	55.9	41.5	2.6
CE 18:1	0.12	0.17	0.2	13.5	0.0	86.5
CE 18:2	0.17	0.19	0.1	4.0	0.0	96.0
CE 18:3	0.37	0.21	0.03	24.0	16.8	59.2
CE 20:3	0.20	0.20	0.2	0.0	0.0	100.0
CE 20:4	0.37	0.20	0.02	0.0	0.0	100.0
CE 20:5	0.38	0.21	0.03	19.3	34.5	46.1
CE 22:6	0.37	0.21	0.03	0.0	0.0	100.0
DAG 34:1	0.39	0.22	0.03	10.8	0.0	89.2
DAG 34:2	0.74	0.23	0.0006	10.6	0.0	89.4
DAG 36:1	0.44	0.21	0.01	7.0	0.0	93.0
DAG 36:2	0.72	0.23	0.0002	7.1	12.3	80.6
LPC 14:0	1.0E-06	0.16	0.5	100.0	0.0	0.0
LPC 16:0	0.19	0.16	0.06	1.1	45.7	53.2
LPC 16:1	0.02	0.10	0.4	100.0	0.0	0.0
LPC 18:0	0.38	0.19	0.002	14.4	41.6	44.0
LPC 18:1	0.05	0.12	0.3	19.6	0.0	80.4
LPC 18:2	0.03	0.14	0.4	0.0	0.0	100.0
LPC 20:3	0.24	0.18	0.04	2.2	0.0	97.8
LPC 20:4	0.54	0.21	0.0001	14.4	24.1	61.6
LPC 20:5	0.27	0.16	0.002	10.9	44.6	44.5
LPC 22:6	0.43	0.18	0.0002	0.0	34.2	65.8
LPE 16:0	0.41	0.19	0.002	0.0	35.2	64.8
LPE 18:0	0.48	0.21	0.0008	11.7	33.3	55.0
LPE 18:1	0.14	0.13	0.07	0.2	16.3	83.5
LPE 18:2	1.0E-06	0.10	0.5	100.0	0.0	0.0
LPE 20:4	0.30	0.18	0.009	26.3	7.6	66.1
LPE 22:6	0.36	0.19	0.003	12.1	41.8	46.1
PC 32:0	0.45	0.20	0.0004	4.1	24.8	71.1
PC 32:1	0.01	0.12	0.5	0.0	0.0	100.0
PC 32:2	1.0E-06	0.16	0.5	0.0	3.9	96.1
PC 34:1	1.0E-06	0.16	0.5	0.0	0.0	100.0
PC 34:2	1.0E-06	0.12	0.5	79.9	20.1	0.0
PC 34:3	0.22	0.19	0.1	0.0	20.3	79.7
PC 34:4	1.0E-06	0.22	0.5	33.3	33.3	33.3
PC 36:1	0.19	0.21	0.2	0.0	26.9	73.1
PC 36:2	0.08	0.18	0.4	81.9	18.1	0.0
PC 36:3	0.15	0.17	0.2	23.8	20.8	55.4
PC 36:5	0.15	0.17	0.2	47.8	36.5	15.7
PC 36:4	0.24	0.17	0.03	0.0	30.7	69.3
PC 38:2	0.37	0.20	0.01	0.0	34.7	65.3
PC 38:3	0.23	0.18	0.05	0.0	1.8	98.2
PC 38:4	0.41	0.18	0.0004	17.4	24.0	58.6
PC 38:5	0.32	0.18	0.006	15.9	43.2	41.0
PC 38:6	0.33	0.18	0.003	13.5	35.7	50.8
PC 40:6	0.38	0.17	0.0003	17.2	33.3	49.5
SM 14:0	1.0E-06	0.11	0.5	33.3	33.3	33.3
SM 16:0	0.42	0.21	0.02	10.5	0.0	89.5
SM 16:1	0.18	0.17	0.08	0.0	30.8	69.2
SM 18:0	0.24	0.20	0.08	0.0	3.9	96.1
SM 18:1	0.44	0.22	0.02	0.0	6.5	93.5
SM 22:0	0.10	0.19	0.4	0.0	35.1	64.9
SM 22:1	0.33	0.22	0.1	0.0	4.8	95.2
SM 24:0	0.24	0.18	0.06	0.0	16.4	83.6
SM 24:1	0.31	0.16	0.0009	0.6	42.9	56.4
TAG 44:1	0.31	0.22	0.1	0.0	0.0	100.0
TAG 46:0	0.21	0.23	0.2	0.0	0.0	100.0
TAG 46:1	0.21	0.22	0.3	0.0	0.0	100.0
TAG 46:2	0.26	0.22	0.2	0.0	0.0	100.0
TAG 48:0	0.23	0.21	0.2	0.0	0.0	100.0
TAG 48:1	1.0E-06	0.17	0.5	0.0	0.0	100.0

TAG 48:2		0.15	0.22	0.5	0.0	0.0	100.0
TAG 48:3		0.38	0.23	0.09	0.0	0.0	100.0
TAG 48:4		0.55	0.23	0.009	19.3	0.0	80.7
TAG 50:1		0.20	0.25	0.4	0.0	0.0	100.0
TAG 50:2		1.0E-06	0.19	0.5	0.0	0.0	100.0
TAG 50:3		0.12	0.22	0.5	0.0	12.5	87.5
TAG 50:4		0.51	0.23	0.02	0.0	15.3	84.7
TAG 50:5		0.79	0.23	0.0003	21.1	13.3	65.6
TAG 52:1		0.50	0.22	0.02	0.0	0.0	100.0
TAG 52:2		0.53	0.22	0.006	7.4	0.0	92.6
TAG 52:3		0.59	0.23	0.004	0.1	21.3	78.7
TAG 52:4		0.61	0.23	0.002	0.0	23.2	76.8
TAG 52:5		0.74	0.23	0.0001	8.3	30.4	61.3
TAG 52:6		0.66	0.22	0.0001	16.2	29.4	54.5
TAG 54:1		1.0E-06	0.18	0.5	0.0	0.0	100.0
TAG 54:2		0.49	0.21	0.004	0.0	6.8	93.2
TAG 54:3		0.44	0.20	0.005	11.8	29.9	58.3
TAG 54:4		0.36	0.20	0.01	14.1	33.3	52.6
TAG 54:5		0.70	0.22	3.0E-05	12.2	30.4	57.4
TAG 54:6		0.84	0.23	4.0E-07	13.8	30.5	55.7
TAG 54:7		0.70	0.22	6.00E-06	13.5	34.7	51.7
TAG 54:8		0.60	0.22	0.0003	17.0	30.1	52.9
TAG 54:9		0.31	0.21	0.08	7.9	13.3	78.8
TAG 56:10		0.67	0.22	0.0004	14.0	25.0	61.0
TAG 56:2		0.28	0.21	0.06	0.0	6.0	94.0
TAG 56:3		0.26	0.18	0.03	0.0	28.3	71.7
TAG 56:4		0.38	0.20	0.01	0.0	38.9	61.1
TAG 56:5		0.59	0.20	1.0E-05	5.5	38.2	56.3
TAG 56:6		0.64	0.21	8.0E-07	8.8	40.8	50.4
TAG 56:7		0.69	0.21	2.0E-07	15.5	35.3	49.2
TAG 56:8		0.68	0.21	6.0E-07	17.3	37.3	45.4
TAG 56:9		0.83	0.22	2.0E-07	18.4	34.8	46.8
TAG 58:10		0.72	0.22	4.0E-07	17.8	34.7	47.5
TAG 58:11		0.67	0.22	8.0E-06	18.0	39.9	42.1
TAG 58:12		0.74	0.23	0.0006	22.7	29.0	48.4
TAG 58:6		0.38	0.21	0.005	6.5	50.7	42.7
TAG 58:7		0.39	0.22	0.006	13.7	42.7	43.6
TAG 58:8		0.62	0.21	4.0E-06	17.9	34.3	47.8
TAG 58:9		0.65	0.21	1.0E-06	20.0	35.4	44.6
TAG 60:12		0.39	0.20	0.002	19.4	44.0	36.6
2-hydroxyglutaric acid		0.67	0.26	0.007	22.3	23.5	54.2
3-hydroxyphenylacetic acid	3-OH-phenylacetate	0.06	0.14	0.3	0.0	79.2	20.8
3-methyladipic acid; pimelic acid	3-methyladipate; pimelate	0.43	0.25	0.05	11.0	31.3	57.7
3-phosphoglyceric acid		1.0E-06	0.22	0.5	0.0	0.0	100.0
aconitate		1.0E-06	0.19	0.5	0.0	70.4	29.6
adenosine diphosphate	ADP	0.16	0.24	0.4	71.4	11.4	17.2
adenosine monophosphate	AMP	3.3E-03	0.08	0.5	35.8	64.1	0.0
adipic acid	adipate	1.0E-06	0.18	0.5	0.0	42.7	57.3
alpha-ketoglutarate	α-ketoglutarate	1.0E-06	0.11	0.5	0.0	65.0	35.0
alpha-hydroxybutyric acid	α-hydroxybutyric acid	0.22	0.24	0.3	0.0	0.0	100.0
aminoadipic acid		0.52	0.27	0.04	18.4	18.9	62.8
beta-hydroxybutyric acid	β-hydroxybutyric acid	0.04	0.17	0.5	0.0	0.0	100.0
citrate		1.0E-06	0.19	0.5	0.0	77.8	22.2
cyclic adenosine monophosphate	cAMP	1.0E-06	0.08	0.5	100.0	0.0	0.0
cystathionine		0.11	0.17	0.2	7.1	13.0	79.9
fructose; glucose; galactose	hexose	1.0E-06	0.21	0.5	0.0	27.1	72.9
fructose-1-phosphate; fructose-6-phosphate; glucose-1-phosphate; glucose-6-phosphate	hexose monophosphate	0.21	0.26	0.5	0.0	37.8	62.2
fumarate; maleate; α-ketoisovalerate	fumarate; MAE; α-keto-IVA	1.0E-06	0.13	0.5	0.0	79.5	20.5

gentisic acid		1.0E-06	0.19	0.5	0.0	73.7	26.3
glucuronate		0.36	0.25	0.06	0.0	38.7	61.3
glycerol-3-phosphate	glycerol-3-P	0.17	0.19	0.1	7.9	46.7	45.4
glycocholate		0.06	0.16	0.3	0.3	13.0	86.7
glycodeoxycholate; glycochenodeoxycholate	glyco-DCA; glyco-CDCA	0.39	0.26	0.1	0.0	0.0	100.0
guanosine diphosphate	GDP	1.0E-06	0.16	0.5	33.3	33.3	33.3
guanosine monophosphate	GMP	0.08	0.15	0.2	14.4	69.7	15.9
hippuric acid		1.0E-06	0.24	0.5	0.0	0.0	100.0
hyodeoxycholate; ursodeoxycholate; chenodeoxycholate; deoxycholate	hyo/urso/cheno-DCA	1.0E-06	0.18	0.5	33.3	33.3	33.3
hypoxanthine		0.24	0.27	0.3	0.0	26.2	73.8
indole-3-propionate		0.33	0.23	0.05	17.6	19.4	63.0
indoxyl sulfate		0.86	0.26	0.0002	3.9	20.7	75.5
inosine		1.0E-06	0.19	0.5	0.0	0.0	100.0
inositol		1.0E-06	0.10	0.5	99.8	0.1	0.1
isocitrate		0.24	0.24	0.2	0.0	52.0	48.0
kynurenine		0.97	0.27	0.0002	11.7	7.5	80.8
lactate		1.0E-06	0.14	0.5	33.3	33.3	33.3
lactose		1.0E-06	0.12	0.5	33.3	33.3	33.3
malate		0.32	0.21	0.01	0.0	39.6	60.4
orotic acid		1.0E-06	0.19	0.5	33.3	33.3	33.3
oxalate		0.04	0.11	0.3	87.8	12.2	0.0
pantothenic acid		1.0E-06	0.16	0.5	0.0	81.7	18.2
phosphocreatine		1.0E-06	0.21	0.5	33.3	33.3	33.3
phosphoenolpyruvic acid		0.12	0.21	0.3	54.6	45.4	0.0
propionic acid		1.0E-06	0.16	0.5	0.0	0.0	100.0
pyridoxate		0.46	0.23	0.005	13.2	28.7	58.1
pyruvate		1.0E-06	0.21	0.5	0.0	0.0	100.0
quinolinic acid		0.68	0.28	0.009	14.1	24.7	61.1
ribose-5-phosphate; ribulose-5-phosphate	ribose-5-P; ribulose-5-P	1.0E-06	0.19	0.5	0.0	100.0	0.0
salicylurate		1.0E-06	0.21	0.5	100.0	0.0	0.0
sorbitol		1.0E-06	0.09	0.5	33.3	33.3	33.3
suberic acid		0.23	0.29	0.4	23.7	43.6	32.6
sucrose		0.18	0.22	0.3	0.0	55.2	44.8
taurocholate		0.34	0.30	0.1	17.5	0.0	82.5
taurodeoxycholate; taurochenodeoxycholate	tauro-DCA; tauro-CDCA	0.66	0.27	0.004	0.6	3.1	96.2
uric acid	urate	0.21	0.22	0.2	0.0	0.0	100.0
uridine		0.27	0.24	0.2	0.0	19.6	80.4
uridine diphosphate	UDP	0.04	0.17	0.4	45.3	54.6	0.0
uridine diphosphate glucose; uridine diphosphate galactose	UPD glucose/galactose	0.09	0.21	0.4	0.0	16.4	83.6
xanthine		0.08	0.14	0.2	7.4	64.4	28.2
xanthurenate		0.46	0.26	0.05	48.1	0.5	51.4

CE: cholesterol ester; DAG: diacylglycerol; LPC: lysophosphatidylcholine; LPE: lysophosphatidylethanolamine; PC: phosphatidylcholine; SM: sphingomyelin; TAG: triacylglycerol

Supplementary Table 3 FHS sample clinical characteristics

Trait	*n*=2,076
Age, years	55 (10)
Women, n (%)	1062 (51)
Prevalent cardiovascular disease, n (%)	49 (2)
Diabetes mellitus, n (%)	137 (7)
Body-mass index, kg/m^2	27.6 (5.0)
Total cholesterol, mg/dl	207 (37)
HDL cholesterol, mg/dl	49 (15)
Systolic blood pressure, mmHg	127 (19)
Diastolic blood pressure, mmHg	75 (10)
Antihypertensive treatment, n (%)	424 (20)
Current smoker, n (%)	396 (19)

Data represent means (SD) unless otherwise noted
